# Evaluation of Genome-Enabled Prediction for Carcass Primal Cut Yields Using Single-Step Genomic Best Linear Unbiased Prediction in Hanwoo Cattle

**DOI:** 10.3390/genes12121886

**Published:** 2021-11-25

**Authors:** Masoumeh Naserkheil, Hossein Mehrban, Deukmin Lee, Mi Na Park

**Affiliations:** 1 Animal Breeding and Genetics Division, National Institute of Animal Science, Cheonan-si 31000, Chungcheongnam-do, Korea; masoumehnaser@gmail.com; 2 Department of Animal Science, Shahrekord University, Shahrekord 88186-34141, Iran; hosseinmehrban@gmail.com; 3 Department of Animal Life and Environment Sciences, Hankyong National University, Jungang-ro 327, Anseong-si 17579, Gyeonggi-do, Korea

**Keywords:** genomic prediction, accuracy, primal cut yields, single-step method, Hanwoo

## Abstract

There is a growing interest worldwide in genetically selecting high-value cut carcass weights, which allows for increased profitability in the beef cattle industry. Primal cut yields have been proposed as a potential indicator of cutability and overall carcass merit, and it is worthwhile to assess the prediction accuracies of genomic selection for these traits. This study was performed to compare the prediction accuracy obtained from a conventional pedigree-based BLUP (PBLUP) and a single-step genomic BLUP (ssGBLUP) method for 10 primal cut traits—bottom round, brisket, chuck, flank, rib, shank, sirloin, striploin, tenderloin, and top round—in Hanwoo cattle with the estimators of the linear regression method. The dataset comprised 3467 phenotypic observations for the studied traits and 3745 genotyped individuals with 43,987 single-nucleotide polymorphisms. In the partial dataset, the accuracies ranged from 0.22 to 0.30 and from 0.37 to 0.54 as evaluated using the PBLUP and ssGBLUP models, respectively. The accuracies of PBLUP and ssGBLUP with the whole dataset varied from 0.45 to 0.75 (average 0.62) and from 0.52 to 0.83 (average 0.71), respectively. The results demonstrate that ssGBLUP performed better than PBLUP averaged over the 10 traits, in terms of prediction accuracy, regardless of considering a partial or whole dataset. Moreover, ssGBLUP generally showed less biased prediction and a value of dispersion closer to 1 than PBLUP across the studied traits. Thus, the ssGBLUP seems to be more suitable for improving the accuracy of predictions for primal cut yields, which can be considered a starting point in future genomic evaluation for these traits in Hanwoo breeding practice.

## 1. Introduction

In beef production systems, carcass value is the main revenue source, and it is determined by both meat quantity and quality. Acceptable carcass and meat quality characteristics are of great importance in the beef industry, as consumers are willing to pay more for higher-quality products [1]. Hanwoo, the predominant beef cattle of Korea, is well known for its rapid growth rate and quality features such as the bountiful marbling, tenderness, texture, flavor, and juiciness of its beef [2]. Recently, there has been growing interest in including not only traits directly related to profitability, like back fat thickness (BFT), carcass weight (CW), eye muscle area (EMA), and marbling score (MS) [3], but also traits related to the composition of the carcass in the Hanwoo breeding program. Thus, cattle breeders need to address carcass composition and quality traits, which determine the attainability of premium prices and consumer acceptance of meat. Primal cut traits have been proposed to characterize the carcass composition in beef cattle [4]. These traits as a potential indicator of cutability have previously shown moderate-to-high heritability estimates in beef cattle [4,5] and are known to be genetically associated with carcass merit [5,6]. Thus, the improvement of primal cut yields is particularly important in Hanwoo because the evaluation of animals via these traits can be an alternative to facilitate decision-making regarding selection for desirable carcass characteristics. However, it is a challenge to improve these traits using traditional breeding approaches, since they are expensive and difficult to measure or it requires the selection candidates to be slaughtered.

The availability of genome-wide marker panels and an increased number of genotyped individuals have enabled improvements in the accuracy of the estimated breeding value (EBV). Recently, the single-step genomic best linear unbiased prediction (ssGBLUP) method has been widely applied in routine genomic evaluation [7,8,9,10]. Single-step genomic BLUP provides the most comprehensive information for genomic evaluation where the phenotypes of genotyped and non-genotyped individuals, pedigree, and genotype data can be considered simultaneously in one step. Several studies have reported that the use of ssGBLUP improves genomic prediction accuracy relative to pedigree-based BLUP or genomic BLUP (GBLUP) for carcass traits [11,12,13,14,15] and linear body measurements in Hanwoo cattle [16], as well as in other beef cattle [17,18,19]. Nonetheless, utilizing primal cut traits to further increase the accuracy of genomic prediction in breeding programs of Hanwoo cattle has not yet been investigated. Therefore, the present study aimed to assess the accuracy, bias, and dispersion of breeding values for primal cut traits using pedigree-based BLUP (PBLUP) and single-step genomic BLUP (ssGBLUP) methods with a linear regression (LR) method [20]; this can provide valuable information for further implementation of genomic selection for the traits under study in Hanwoo cattle.

## 2. Materials and Methods

### 2.1. Animals and Phenotypes

The dataset used in this study was derived from 3467 Hanwoo steers born between 2008 and 2017 at the Hanwoo Experiment Station, National Institute of Animal Science (NIAS), Rural Development Administration, South Korea. All steers were slaughtered at approximately 24 months of age. The pedigree data containing 18,809 animals was used. The phenotypic data of the primal cut yields were recorded in kilograms and composed of both unique and composite meat cuts from the forequarters and hindquarters. The 10 traits analyzed were bottom round, brisket, chuck, flank, rib, shank, sirloin, striploin, tenderloin, and top round; the locations of each cut on the carcass are illustrated in Figure S1. Summary statistics for each trait are given in Table 1. 

### 2.2. Genotypes and Quality Control 

A total of 3745 animals (2957 steers and 788 bulls) were genotyped (animal and SNP call rate of >90%) with the Illumina BovineSNP50K BeadChip (Illumina Inc., San Diego, CA, USA), including 52,791 SNPs on the 29 chromosomes. SNPs were excluded if they had a minor allele frequency less than 0.01 (8783 SNPs) and extreme deviation from the Hardy–Weinberg equilibrium (defined as the maximum difference between the observed and expected heterozygosity) greater than 0.15 (21 SNPs). For the genotyped individuals, SNPs with missing genotypes were imputed using FImpute V3 software [21], after which, genotypes for 43,987 SNPs remained for subsequent analyses.

### 2.3. Statistical Analyses

#### 2.3.1. Variance Components Estimation

The estimation of variance components and heritabilities were performed through classical Bayesian inference under a single-trait pedigree-based animal model in gibbsf90test software [22] as follows:

(1) y=Xb+Zu+e

where **y** is the vector of observations for the trait of interest; **b** is the vector representing the fixed effects that included the slaughter date (180 levels) and slaughter age (days from birth to slaughter) as covariates for carcass traits; **X** is an incidence matrix related to the fixed effects; **Z** is an incidence matrix related to the random genetic additive effects; **u** is the vector of random genetic additive effects; and **e** is the vector of random residual effects. Random effects were assumed to be distributed as **u** ~ N (0, **A**σ_a_^2^) and **e** ~ N (0, **I**σ_e_^2^), where **A** is the numerator relationship matrix, **I** is the identity matrix, σ_a_^2^ is the additive genetic variance, and σ_e_^2^ is the residual variance.

For this study, Markov Chain Monte Carlo (MCMC) chains comprising 550,000 cycles with the first 50,000 iterations discarded as burn-in (with a thinning interval of 50) were implemented to estimate variance components and heritability as the posterior means of the corresponding sampled values. In addition to these criteria, the chain convergence was assessed by visual inspection. 

Moreover, the coefficient of genetic variation (CV_g_) for each trait was considered as the genetic standard deviation divided by the mean value for the trait of interest [23].

#### 2.3.2. Methods

The statistical methods used in this study to estimate breeding values were a traditional BLUP (PBLUP) method with pedigree-based relationship matrix and a single-step GBLUP (ssGBLUP) method with a combined relationship matrix constructed from genotyped and non-genotyped individuals and pedigree information. The analyses were performed using gibbsf90test software from BLUPF90 family [22]. The estimated breeding value (EBV) was obtained through a traditional genetic evaluation performed without genomic information using Equation (1). The ssGBLUP is a modification of the BLUP model, in which the numerator relationship matrix **A**^−1^ is replaced by **H**^−1^ [7] as follows:

(2) $$H^{-1}=A^{-1}+\left[\begin{array}{cc} 0 & 0\\ 0 & {\left(0.95G+0.05A_{22}\right)}^{-1}-A_{22}^{-1} \end{array}\right]\;$$

where **A**_22_ is the numerator relationship matrix for genotyped animals and **G** is the genomic relationship matrix [24], which was obtained using preGSf90 software [25].

### 2.4. Validation and Method Comparison

The two methods were compared in terms of population accuracy, dispersion, and bias. Validation was performed to evaluate these methods using the LR method as described by Legarra and Reverter [20]. In brief, the LR method estimates the accuracy, bias, and dispersion based on a comparison of EBV/GEBV obtained with less information (partial dataset) and EBV/GEBV derived with more information (whole dataset) for the same group of individuals. The focal individuals were defined as steers born in 2016 and 2017 (605 animals) in the validation data for primal cut traits. Hence, the EBV/GEBV for focal animals in the whole and partial dataset with the two methods were estimated. In the partial data, it was assumed that the phenotypes of focal animals are unknown, and only the genotypes and pedigree information were available. The expectation of EBV/GEBV accuracy from the partial dataset is $\rho_{w,p}=\sqrt{\frac{\mathrm{cov}\left({\hat{u}}_{w},{\hat{u}}_{p}\right)}{\left(1+\;\bar{F}+2\;\bar{f}\right)\sigma_{u,\infty }^{2}}}$, where $\;\bar{F}$ is the average inbreeding coefficient, 2$\;\bar{f}$ is the average relationship between individuals, ${\hat{u}}_{w}$(${\hat{u}}_{p}$) is the vector of estimated breeding values for focal animals using the whole (partial) dataset [20], and $\sigma_{u,\infty }^{2}$ is the genetic variance at equilibrium in a population under selection, which is estimated via the Gibbs sampling approach proposed by Sorensen et al. [26]. In addition, the estimator that was also used in this study is $\frac{1}{\rho_{PBLUP,ssGBLUP}}$, which represents the relative increase in accuracy from the PBLUP method to ssGBLUP in the whole or partial dataset [20].

Thus, $\frac{1}{\rho_{PBLUP,ssGBLUP}}-1$ is superior in accuracy when using ssGBLUP compared to the PBLUP model for all the traits when the evaluation method changes from PBLUP to ssGBLUP. It is expected that genomic methods increase the accuracy of predictions as compared with pedigree-based evaluations for steers. Furthermore, the inverse of correlation between ${\hat{u}}_{p}$ and ${\hat{u}}_{w}$ (i.e., $\frac{1}{\rho_{p,w}}$) represents the gain in accuracy from a partial to whole dataset by adding phenotypic information to genetic evaluations.

The expected bias was defined as the difference between the means of EBV/GEBV in the partial and whole datasets, $\mu_{w,p}=\bar{{\hat{u}}_{p}}-\bar{{\hat{u}}_{w}}$. If the evaluation is unbiased, the expected value of this estimator is zero. The estimator of dispersion of the breeding value was measured as the regression coefficient of ${\hat{u}}_{w}$ on ${\hat{u}}_{p}$, $b_{w,p}=\frac{\mathrm{cov}\left({\hat{u}}_{w},{\hat{u}}_{p}\right)}{var\left({\hat{u}}_{p}\right)}$. The expected value of this estimator is 1 if there is no over- or under-dispersion of breeding values [20].

## 3. Results

### 3.1. Descriptive Analysis and Estimation of Variance Components

The descriptive statistics for the 10 primal cut yields are given in Table 1. The mean values of these traits ranged from 6.04 to 57.55 kg with a standard deviation between 0.76 and 7.53. The coefficients of variation ranged from 11.89 to 25.72%, indicating considerable phenotypic variation of the studied traits in the Hanwoo cattle. The heritability estimates and variance components for all traits are shown in Table 2. The estimated variance components display that the traits of interest are moderate to highly heritable, with the highest (0.52 ± 0.06) and the lowest heritability (0.21 ± 0.04) found for top round and chuck, respectively.

### 3.2. Comparisons of Prediction Accuracy, Bias, and Dispersion between Pedigree-Based BLUP and ssGBLUP

**Figure 1:**
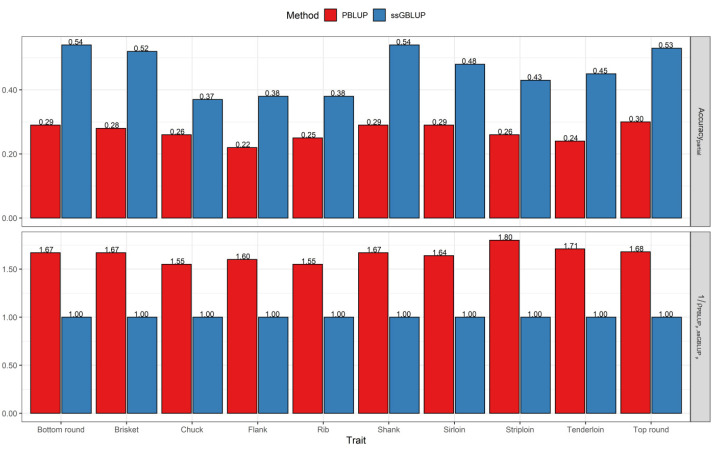
Accuracy of breeding values obtained using the PBLUP and ssGBLUP models for primal cut yields with the partial dataset.$\;\frac{1}{\rho_{PBLUPp,ssGBLUPp}}$: The relative increase in accuracy from PBLUP to the ssGBLUP model on the partial dataset.

**Figure 2:**
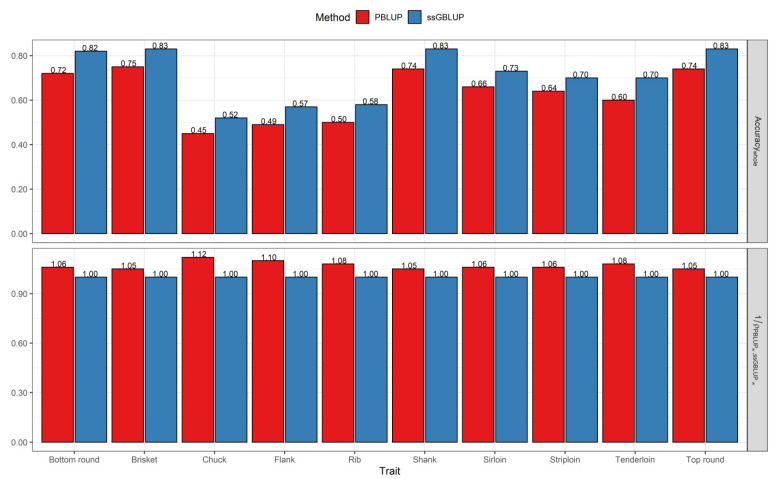
Accuracy of breeding values obtained using the PBLUP and ssGBLUP models for primal cut yields with the whole dataset.$\;\frac{1}{\rho_{PBLUPw,ssGBLUPw}}$: The relative increase in accuracy from PBLUP to the ssGBLUP model on the whole dataset.

The population accuracies for the primal cut yields obtained via the PBLUP and ssGBLUP methods using the partial and whole datasets are presented in Figure 1 and Figure 2. The results show that the accuracies of breeding values from the ssGBLUP method were considerably higher than those from PBLUP for the analyzed traits, regardless of considering a partial or whole dataset. The accuracies for PBLUP ranged from 0.22 to 0.30 and those for ssGBLUP ranged from 0.37 to 0.54 using the partial dataset. The average predictive accuracies were 0.26 for PBLUP and 0.46 for the ssGBLUP method across all traits. In addition, the estimator $\frac{1}{\rho_{PBLUP,ssGBLUP}}-1$, indicating the average value of improved accuracy by switching from PBLUP to ssGBLUP, was 65% across the 10 traits due to the addition of genomic information when a partial dataset was used (Figure 1). 

When the whole dataset for the prediction of breeding values was added in the analyses, accuracies ranged from 0.45 to 0.75 (average 0.62) for PBLUP and from 0.52 to 0.83 (average 0.71) for the ssGBLUP model, indicating an improvement in accuracy with ssGBLUP compared with PBLUP for the studied traits (Figure 2). In other words, the ssGBLUP was superior to PBLUP, with an average relative gain of 7% across all traits when using the whole dataset (Figure 2).

**Figure 3:**
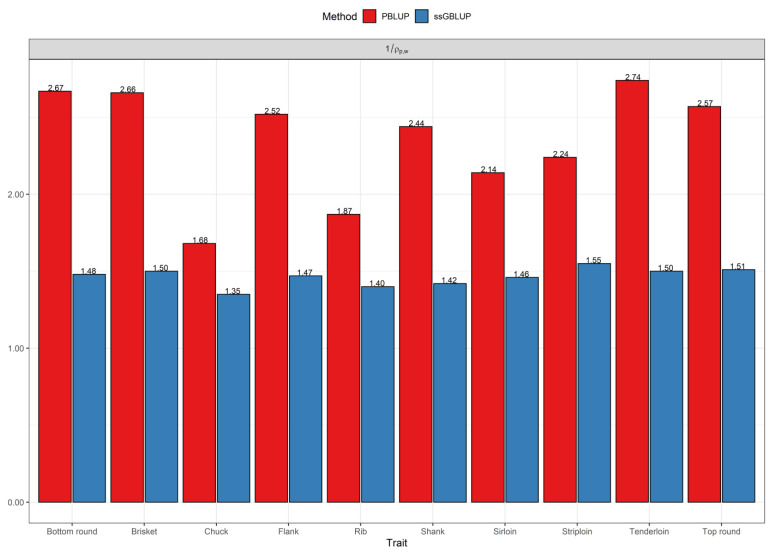
The relative increase in the accuracy of breeding values from the partial dataset to the whole dataset ($\frac{1}{\rho_{p,w}}$) using PBLUP and ssGBLUP models for primal cut yields.

The results obtained with a partial dataset were compared with those from the whole dataset in terms of accuracy in both the pedigree and genomic methods. The results show that the population accuracies using the whole dataset were higher than those derived from a partial dataset. Figure 3 shows that the accuracy across the studied traits using the whole dataset was two times than those with the partial dataset in the PBLUP method, while the accuracy was improved by 46% on average for the ssGBLUP method from the partial to the whole dataset.

**Figure 4:**
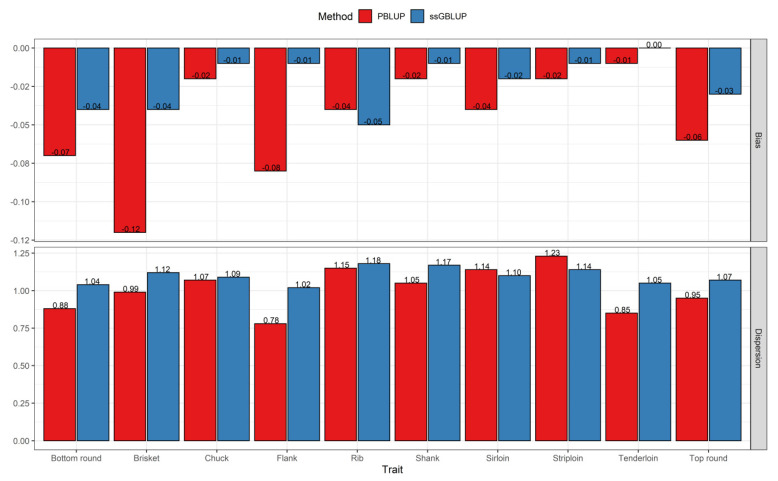
Bias and Dispersion of breeding values obtained using the PBLUP and ssGBLUP models for primal cut yields on the partial dataset.

Regarding the bias, predictions from the ssGBLUP method displayed less bias than predictions from the PBLUP method for the traits under study, except for rib. The average values varied from −0.12 to −0.01 for PBLUP and from −0.05 to 0 for the ssGBLUP method across all traits (Figure 4). Among the primal cut traits, the predictions from ssGBLUP were unbiased for tenderloin; following that, chuck, flank, shank, and striploin were slightly less biased or closer to zero compared to the other remaining traits. Moreover, the value for the estimator of dispersion for all traits ranged from 0.78 to 1.23 (average absolute deviation from 1 equal to 0.12) for PBLUP and from 1.02 to 1.18 (average absolute deviation from 1 equal to 0.10) for the ssGBLUP method (Figure 4). Generally, these results indicate that ssGBLUP leads to less over- or under-dispersion compared with PBLUP in the analyzed dataset.

## 4. Discussion

The current study is the first attempt to compare pedigree-based BLUP (PBLUP) and single-step genomic BLUP (ssGBLUP) methods for prediction accuracy regarding primal cut yields in Hanwoo cattle. To maximize the profitability of the beef cattle industry, selection based on genomic information has become an effective tool in breeding programs. According to our results, primal cut yields are moderately to highly heritable traits, comparable to those estimated in Hanwoo cattle [5], Simmental cattle [27], Chianina cattle [28], Irish cattle [4,6], and U.K. beef cattle [29]. For instance, compared to our results, Choi et al. [5] estimated higher heritability for bottom round (0.66), rib (0.35), sirloin (0.60), striploin (0.64), tenderloin (0.41), and top round (0.62); lower heritability for brisket (0.21), flank (0.21), and shank (0.35); and slightly similar heritability for chuck (0.19) in Hanwoo cattle. This difference might result from the discrepancy in the number of records measured (3467 vs. 920 records) and the units of measurement data (kilograms vs. percent of carcass weight). Furthermore, the existence of sufficient genetic variation indicates that improvements in these traits through genetic selection are feasible. In a recent study, we observed the favorable and moderate to high genetic correlations between the primal cut yields and three composite traits of primal cuts based on their retail value (high-value cuts, medium-value cuts, and low-value cuts) with carcass traits available in the Hanwoo selection index, indicate that these traits can be considered as a selection objective in the Hanwoo breeding scheme [30]. A similar conclusion was drawn by Pabiou et al. [31], who reported that four groups of wholesale cut weights predicted using video image analyses (i.e., very high value cuts, high value cuts, medium value cuts, and low value cuts) had moderate genetic correlations with carcass weight in Irish steers. Given that primal cuts yields in beef cattle are known to be heritable traits, evidence exists, although based on a limited study population [4,6], which shows that these traits could be useful in multi-trait genetic evaluations targeting the improvement in the accuracy of selection for primal carcass cut weights and, by extension, overall carcass merit.

In this study, estimates of the accuracy, bias, and dispersion of primal cut yields were obtained via evaluation models that used only pedigree or a combination of pedigree and genomic relationship matrices using the LR method. Our results demonstrated that the ssGBLUP model outperformed PBLUP for all 10 traits in either a partial or whole dataset. A shift in the average accuracy from 0.26 to 0.46 was obtained by changing from a PBLUP to ssGBLUP method across the studied traits using the partial dataset. Similarly, the use of the whole dataset in analyses led to an average increase in accuracy from 0.62 to 0.71 by moving the model from PBLUP to ssGBLUP for all the primal cut yields. In other words, when genomic information was included in the analyses, the average improvements in the accuracy were + 20 and + 9 percentage points using partial and whole datasets, respectively, compared with pedigree-based BLUP due to the capture of variation in Mendelian sampling [32,33]. Furthermore, the increase in accuracy in genomic prediction depends upon the quality and veracity of the available information, complete or incorrect pedigree, number of phenotyped/genotyped animals, and heritability of traits [34]. It has also been shown that the use of all phenotyped animals, pedigree, and genomic information simultaneously in the ssGBLUP model provides better predictability compared to the pedigree-based model, which could also be explained by better relationships [24,35]. Hence, our results showed that using genomic prediction is relatively more beneficial for all evaluated traits, thereby enabling a significant increase in the rate of genetic gain [36].

Among the studied traits, ssGBLUP provided a substantially higher prediction accuracy than the PBLUP method for bottom round, shank, top round, and brisket compared with the other traits. It is reasonable to assume that this higher accuracy could be due to the heritability of the traits of interest, which were 0.50, 0.50, 0.52, and 0.51 herein for bottom round, shank, top round, and brisket, respectively (Table 2). The importance of heritability on the accuracies of estimated breeding values is shown in Figure S2, where the regression coefficients of accuracy (in the partial data) on the heritabilities were significantly positive regardless of the method. As expected, the superiority of ssGBLUP over PBLUP was more obvious for the traits with higher heritability, suggesting that heritability can be used as a measure to evaluate the accuracy of the target traits in genomic evaluation for a breeding program. Similar findings were found by Bolormaa et al. [37], who reported that the most accurate predictions were observed for the traits with highest heritability. In addition, another study showed that higher prediction accuracies were obtained for ADG and DMI, which had the highest heritabilities (h^2^ = 0.39 and h^2^ = 0.43, respectively) among the feed efficiency traits in Nellore cattle [38]. 

Concerning the ratio of the superiority of ssGBLUP over the pedigree-based method, the performance of the estimation of the ratio was improved by the added genomic information in ssGBLUP compared with the PBLUP method across all traits. The estimated value of the ratio indicates the increase in accuracy from pedigree to genomic predictions in both partial and whole datasets. The results indicate that the relative gain in the accuracy of GEBV with ssGBLUP was 65% using the partial dataset, whereas the ssGBLUP method was superior to PBLUP when the whole dataset was used, with an average increase or relative gain of 7% across all traits. In fact, these findings indicate the positive impact of genomic information on the genetic evaluations when no phenotypic data are available for the candidate animals or even when phenotypes are available on individuals; the addition of genomic information in the analysis can considerably improve the accuracy of prediction relative to pedigree-based models.

According to our results, the increase in accuracy from partial to whole dataset ($\frac{1}{\rho_{p,w}}$) was over two times for the pedigree-based evaluation compared to the genomic-based method (Figure 3). This indicates that the accuracy of predictions for the genomic method compared with the pedigree-based method is less affected by the use of the whole dataset, which is in agreement with the literature [20,39,40,41]. For instance, Granado-Tajada et al. [40] showed that adding phenotypes increased accuracy by double and 81% using the pedigree evaluation for two strains of dairy sheep, namely Latxa Cara Rubia (LCR) and Latxa Cara Negra from Euskadi (LCNEUS), respectively, while this estimator was 96% and 78% using a genomic method for LCR and LCNEUS, respectively. Moreover, in another study on dairy sheep (Manech Tête Rousse breed), Macedo et al. [41] exhibited an increase in accuracy of 78% for the PBLUP method and 51% for the ssGBLUP method by adding phenotypic information of focal individuals to the partial dataset. Similarly, Bermann et al. [39] reported that the accuracies based on the LR method were 0.45 for BLUP and 0.76 for ssGBLUP in the simulated data, whereas those obtained with the real data (chicken mortality) were 0.41 and 0.47 for BLUP and ssGBLUP, respectively. These authors also demonstrated that moving from the partial to the whole dataset resulted in a 17% and 20.4% gain in accuracy based on the pedigree evaluation and a 14% and 9.8% increase for the genomic-based evaluation in real and simulated data, respectively.

These findings were also in concordance with those reported by Mehrban et al. [14], who applied the LR method to evaluate carcass traits in Hanwoo cattle using four evaluation models and showed that using genomic information and additional phenotypic information from highly genetically correlated traits is a suitable tool to improve the prediction accuracy. 

Consistent with our results, several previous studies have proved the superiority of ssGBLUP over traditional BLUP approaches in various livestock since it was proposed [7,18,32,33,42,43,44,45,46,47,48,49,50]. Moreover, in Hanwoo cattle studies, the accuracies obtained from single-step models were found to be higher than those from pedigree-based and multi-step methods for carcass traits [11,12,13,14,15,51] and body measurement traits [16].

This estimator was proposed by Legarra and Reverter [20], and it is an estimator of the ratio between accuracies based on pedigree and genomic evaluations in partial and whole datasets. They also highlighted that the correlation between predictions obtained with partial and whole datasets is not a measure of accuracy, but an estimator of the ratio between accuracies. In fact, the advantage of this method is the simplicity of its application, comparing the EBV/GEBV estimated from less information (partial dataset) with the EBV/GEBV from data containing more information (whole dataset) for the same individuals in different evaluations. 

In addition to the prediction accuracy, the bias and estimator of dispersion are other important indicators when comparing different prediction methods in breeding programs. Bias, for which the desired value is zero, refers to the difference between the mean of EBV in partial and whole dataset. In general, predictions obtained via the single-step approach by adding genomic information showed less bias compared with those obtained from the pedigree-based method across the studied traits, except for rib. The dispersion can be defined as the value of the slope of the regression of breeding values estimated with whole dataset on breeding values estimated using partial dataset, and it has an expected value of 1. Dispersion estimates less than or greater than 1 indicate an overestimation (inflation) or underestimation (deflation), respectively. The results showed that the average absolute deviation of dispersion from 1 was less for ssGBLUP than for the PBLUP method. Therefore, the inclusion of all information available in the evaluation simultaneously reduced the overestimation and underestimation of the breeding values [52], and the single-step genomic approach is able to partially account for pre-selection, as expected [53]. It was previously shown that inflation or deflation of predictions could result in incorrect comparisons between animals of different generations and inaccurate genetic trend estimates [54]. 

Consequently, the results of the current study provide a comprehensive analysis of accuracy, bias, and dispersion for primal cut yields using the LR method, which can be applied to any model and any data structure [20,55]. Our results demonstrated that ssGBLUP generally provided a higher accuracy than the PBLUP method for primal cut yields in Hanwoo cattle that could have a practical applicability for breeding programs.

## 5. Conclusions

Our aim was to investigate two methods, pedigree-based BLUP and ssGBLUP, for 10 primal cut yields using the LR method in terms of population accuracy, bias, and dispersion in Hanwoo cattle. The results indicated that the ssGBLUP model yielded considerably higher accuracy than the PBLUP model for all the studied traits. Concerning the bias and dispersion, predictions from the ssGBLUP method had less bias and a value of dispersion closer to 1 compared to PBLUP across the primal cut yields. It is worthy to note that the inclusion of all pedigree, phenotypic, and genomic information simultaneously in the ssGBLUP led to a 65% (7%) higher accuracy on average than PBLUP in primal cut yields using the partial (whole) dataset. Therefore, it seems that using ssGBLUP is a promising approach in future genomic evaluations targeting the improvement of weight in the more valuable primal cuts, consequently increasing the profitability of the Hanwoo beef production system.

## Figures and Tables

**Table 1:** Summary statistics for the primal cut yields in Hanwoo cattle.

Trait (Unit)	No. of Records	Mean (SE)	Min.	Max.	SD	CV (%)
Bottom round (Kg)	3467	32.99 (0.07)	16.6	49.6	3.92	11.89
Brisket (Kg)	3466	23.76 (0.05)	12.6	38.6	3.01	12.67
Chuck (Kg)	3463	14.61 (0.06)	6.7	34.8	3.76	25.72
Flank (Kg)	3465	28.29 (0.08)	12.5	50.3	4.83	17.08
Rib (Kg)	3467	57.55 (0.13)	21.7	89.3	7.53	13.09
Shank (Kg)	3466	14.66 (0.03)	9	21.7	1.77	12.09
Sirloin (Kg)	3465	34.23 (0.07)	16.8	50.7	4.11	12.02
Striploin (Kg)	3465	7.85 (0.02)	4.3	12.4	1.17	14.96
Tenderloin (Kg)	3466	6.04 (0.01)	3	9	0.76	12.65
Top round (Kg)	3467	20.22 (0.04)	10.5	30.2	2.43	12

SE, standard error; SD, standard deviation; CV, coefficient of variation.

**Table 2:** Estimates of heritability (h^2^), additive genetic variance (σ^2^_a_), residual variance (σ^2^_e_), and coefficient of genetic variation (CV_g_) for primal cut yields using the pedigree-based animal model in Hanwoo cattle.

Trait	h^2^	σ^2^_a_	σ^2^_e_	σ^2^_p_	CV_g_(%)
Bottom round	0.50 (0.06)	5.47 (0.73)	5.41 (0.59)	10.87 (0.30)	7.09
Brisket	0.51 (0.06)	3.17 (0.42)	3.08 (0.34)	6.25 (0.18)	7.49
Chuck	0.21 (0.04)	1.82 (0.38)	6.64 (0.36)	8.46 (0.22)	9.23
Flank	0.29 (0.05)	4.61 (0.86)	11.58 (0.77)	16.18 (0.42)	7.59
Rib	0.27 (0.05)	9.58 (1.93)	27.18 (1.75)	37.04 (0.96)	5.38
Shank	0.50 (0.06)	1.10 (0.15)	1.11 (0.12)	2.20 (0.06)	7.15
Sirloin	0.42 (0.06)	5.26 (0.78)	7.20 (0.65)	12.46 (0.34)	6.70
Striploin	0.39 (0.06)	0.31 (0.05)	0.50 (0.04)	0.81 (0.02)	7.09
Tenderloin	0.34 (0.05)	0.14 (0.02)	0.27 (0.02)	0.42 (0.01)	6.19
Top round	0.52 (0.06)	2.22 (0.29)	2.07 (0.23)	4.29 (0.12)	7.37

The numbers in parentheses are the standard deviations of posterior densities.

## Data Availability

The data that support the findings of this study are available at [(http://www.ekape.or.kr (accessed on 11 February 2021)) and (http://www.limc.co.kr (accessed on 11 February 2021))].

## Supplementary Materials

The following are available online at https://www.mdpi.com/article/10.3390/genes12121886/s1, Figure S1: Location of 10 carcass primal cut yields in Hanwoo cattle; Figure S2: The regression coefficients of accuracy on the her-itabilities of traits in the partial dataset. The grey zone is the 95% confidence interval.
