# Supplementary material for: Evaluation of Genome-Enabled Prediction for Carcass Primal Cut Yields Using Single-Step Genomic Best Linear Unbiased Prediction in Hanwoo Cattle
Source: Genes (Basel). 2021 Nov 25;12(12):1886. doi: 10.3390/genes12121886 (PMC8701981; doi:10.3390/genes12121886)
Supplement: Supplementary file 1 [file genes-12-01886-s001.zip › genes-1438814-supplementary.pdf]

Supplementary Material

Supplementary Figures:

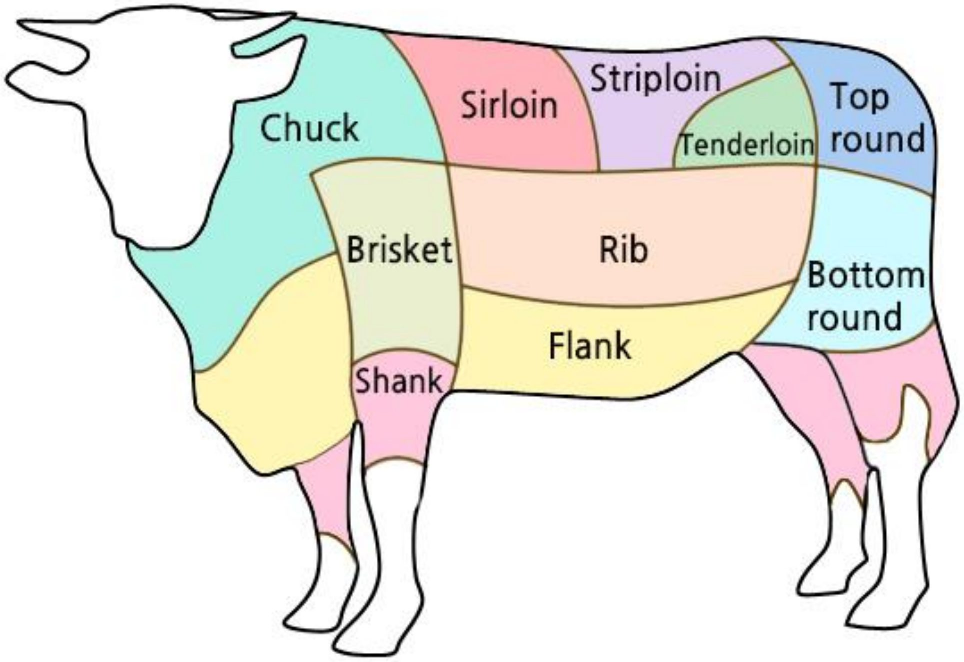

Figure S1. Location of 10 carcass primal cut yields in Hanwoo cattle.

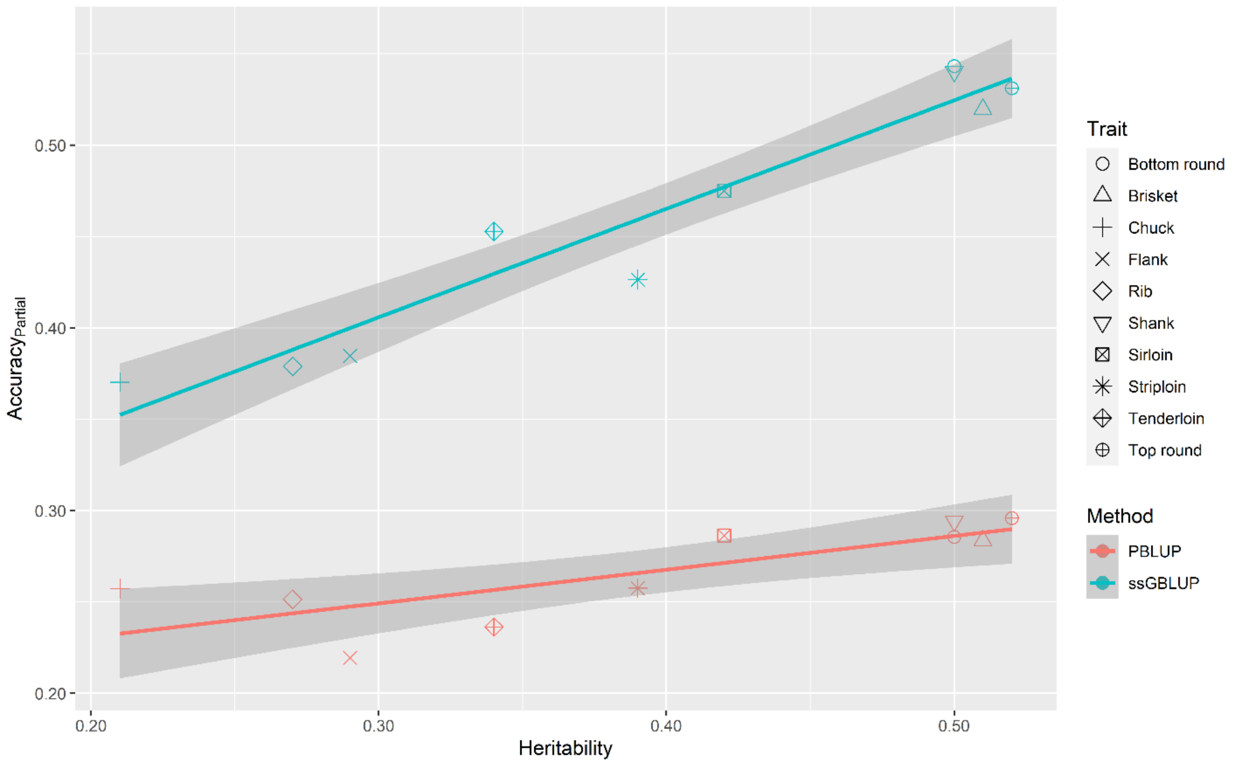

Figure S2. The regression coefficients of accuracy on the heritabilities of traits in the partial dataset.

The grey zone is the 95% confidence interval.
